# Antenatal Vitamin C differentially affects lung development in normally grown and growth restricted sheep

**DOI:** 10.1038/s41390-025-03828-1

**Published:** 2025-02-25

**Authors:** Erin V. McGillick, Sandra Orgeig, Beth J. Allison, Kirsty L. Brain, Youguo Niu, Nozomi Itani, Katie L. Skeffington, Andrew D. Kane, Emilio A. Herrera, Dino A. Giussani, Janna L. Morrison

**Affiliations:** 1 Early Origins of Adult Health Research Group, Health and Biomedical Innovation, Adelaide, Australia; 2 UniSA: Clinical and Health Sciences, University of South Australia, Adelaide, Australia; 3 Department of Physiology, Development & Neuroscience, University of Cambridge, Cambridgeshire, Cambridge, United Kingdom; 4 Programa de Fisiopatología, Instituto de Ciencias Biomédicas, Facultad de Medicina, Universidad de Chile, Santiago, Chile

## Abstract

**Background:**

Chronic hypoxemia is a common cause of fetal growth restriction and can have significant effects on the developing fetal lung. Maternal antioxidant treatment in hypoxic pregnancy protects against offspring cardiovascular dysfunction. The effects of antenatal antioxidants on lung development in the chronically hypoxic growth restricted fetus is unknown.

**Methods:**

We investigated the effect of maternal daily Vitamin C (200 mg/kg i.v. vs. Saline) for a month in late gestation on molecular markers regulating lung maturation between normoxic normally grown and hypoxic growth-restricted fetal sheep. Chronic fetal hypoxia and fetal growth restriction were induced by exposure to maternal chronic hypoxia (10% O_2_ vs. Normoxia=21% O_2_) from 105–138 d gestation (term=145 d).

**Results:**

The data show a differential effect of antenatal Vitamin C treatment on regulation of genes involved in surfactant maturation, sodium movement and hypoxia signaling. Limited responsiveness to antenatal Vitamin C exposure in the lung of the hypoxic fetus, compared to responsiveness to antenatal Vitamin C in the normoxic fetus, suggests a maximal upregulation of the molecular signaling pathways in response to the chronic hypoxic insult alone.

**Conclusion:**

We provide molecular insight into the heterogeneity of antenatal Vitamin C treatment on development of the normoxic and growth restricted hypoxic fetal lung.

**Impact:**

- The effect of maternal Vitamin C on molecular markers of lung maturation between normoxic normally grown and hypoxic growth restricted fetal sheep was unknown.
- We show a differential effect of Vitamin C with a greater increase in molecular markers of lung maturation in normoxic compared with hypoxic fetuses.
- Limited responsiveness in the hypoxic fetal lung is likely due to maximal upregulation by the hypoxic insult alone, thus added exposure to Vitamin C is unable to upregulate the system further.
- The work highlights the need to understand differential effects of antenatal interventions in healthy and complicated pregnancy, prior to clinical translation.

## Introduction

Fetal lung development is under control of oxygen availability, hormones, molecular signaling (including growth factors) and structural/mechanical factors.^1–5^ Suboptimal intrauterine environments affect the development of the fetal lung, thereby influencing the risk of respiratory complications at birth and susceptibility to lung disease in later life.^6–9^ Understanding how pregnancy complications, such as fetal growth restriction, affect the developing lung and optimizing therapies to overcome the effects of suboptimal *in utero* environments to improve newborn outcomes is critically important.

Fetal growth restriction affects approximately 10% of pregnancies worldwide and most commonly occurs due to reduced oxygen and nutrient transport to the developing fetus.^5,10^ Fetal growth restriction has consequences on fetal growth and organ development that can have both direct and indirect effects on physiology leading to increased morbidity and mortality in early and later life.^11–13^ Altered oxygen availability and oxidative stress can have individual and synergistic effects on molecular and structural regulation of fetal lung development.^6^ In normal pregnancy, endogenous antioxidant defense mechanisms mature across late gestation in preparation for exposure to the relative hyperoxic air-breathing environment at birth.^14–17^ However, in pregnancies complicated by oxidative stress, insufficient fetal antioxidant defenses to scavenge the enhanced free radical production at the cellular level can lead to poorer infant outcomes.^12,15,18–21^

Heterogeneity in the effects of antioxidants in pregnancy is well known. For instance, in humans, deficiency of antioxidant Vitamin C during pregnancy is associated with increased preterm premature rupture of the membranes.^22,23^ Conversely, maternal supplementation with the antioxidants Vitamins C and E, to reduce the risk of pre-eclampsia, increased the risk of babies being born small^24^ and had effects on markers of oxidative stress and placental function.^25^ Follow up of these babies also demonstrated no longer term benefits on asthma at 2 years of age.^26^ In hypoxic pregnancy in sheep and rats, maternal treatment with Vitamin C protected against fetal growth restriction and the programming of cardiovascular dysfunction in the adult offspring.^12,27–30^ Clinically, antenatal Vitamin C treatment to pregnant smokers improved lung function in newborns (ratio of the time to peak tidal expiratory flow to expiratory time [TPTEF:TE] and passive respiratory compliance per kilogram,^31^) increased forced expiratory flow at 3 and 12 months of age,^32,33^ and reduced wheeze at 1 year of age. More recently, antenatal Vitamin C supplementation has been shown to ameliorate changes associated with maternal smoking in placental DNA methylation and gene expression of pathways associated with placental function and lung function/wheeze.^34^ This diversity in effects is not unique to antioxidant treatment in pregnancy, with many documented differences in the effects of other pharmacological interventions in the healthy versus growth-restricted fetus, including maternal treatment with antenatal glucocorticoids, sildenafil and melatonin.^7,12,35–43^ Therefore, there is a clear fundamental need for pre-clinical investigation of potential therapeutics in models of complicated pregnancy with heterogeneous causes to ensure that interventions have the desired molecular effects and do not cause unintended harm to individuals.

While clinical studies have provided insights into the heterogeneous incidence and risk of growth restricted newborns experiencing respiratory complications,^7^ they are unable to provide detailed mechanistic insights into factors regulating altered lung development. We have previously demonstrated that exposure to chronic fetal hypoxia^44^ and antenatal antioxidant treatment in normal pregnancy^45^ both have significant effects on molecular regulation of fetal lung maturation. Specifically, exposure to maternal chronic hypoxia increased expression of genes regulating lung liquid reabsorption (sodium and water movement), surfactant maturation and lipid transport and hypoxia signaling.^44^ Antenatal Vitamin C exposure in normal pregnancy increased fetal lung gene expression of antioxidant enzymes, hypoxia signaling, sodium movement, surfactant maturation and airway remodeling.^45^ In this study, we have compared the effects of maternal treatment with antioxidant Vitamin C on lung development between the normoxic normally grown fetus and the hypoxic growth restricted fetus. The study was carried out in sheep, as sheep and humans share similar prenatal milestones of lung development.^46^ We used a dosing regimen of Vitamin C previously validated to protect against oxidative stress in hypoxic pregnancy in this species.^27^ We focused on molecular pathways involved in normal fetal lung development, including surfactant maturation, lung liquid movement, glucocorticoid signaling and hypoxia signaling.

## Methods

All procedures were approved by the University of Cambridge Ethical Review Board and were performed in accordance with the UK Animals (Scientific Procedures) Act 1986. In this study, 34 pregnant Welsh mountain ewes carrying singleton pregnancies were used.

### Surgery and experimental protocol

At 100 ± 1 days gestation (term, ~145 d), all ewes underwent a sterile laparotomy under general anesthesia (1.5–2.0% isofluorane in 60:40 O_2_:N_2_O) to determine fetal sex and catheterization of the maternal femoral artery and vein, as previously described.^27,47,48^ To control for sex differences only male fetuses were included in this study. Female fetuses were assigned to a postnatal study^49^. At 105 d gestation (when the fetal lung is in the canalicular phase of development; equivalent to ~21 weeks human gestation, term = 40 weeks),^50,51^ ewes were randomly assigned to one of four experimental groups. Pregnant ewes assigned to the hypoxic group (*n* = 17) were housed in bespoke isobaric hypoxic chambers (Telstar Ace, Dewsbury, West Yorkshire, UK) from 103 d gestation under normoxic conditions and exposed to ~10% O_2_ from 105 d gestation by altering the incoming inspirate mixture as previously established.^48^ Ewes allocated to the normoxic group (*n* = 17) remained housed in individual floor pens of the same size as the hypoxic chambers and with access to the same nutritional regimen for the duration of the experimental protocol.^27^ While the Control ewes were not housed in the same chambers as the hypoxic group, we controlled for as many factors as possible, including the pen floor surface area, humidity, temperature, noise levels and the feeding regimen.^48^ Critically, animals in both groups were always able to see other sheep, thereby minimizing stress. Within the hypoxic chambers, the inspirate air mixture was passed via silencers able to reduce noise levels within the hypoxic chamber laboratory (76 dB(A)) and inside each chamber (63 dB(A)) to values lower than those necessary to abide by the Control of Noise at Work Regulations. This not only complied with human health and safety and animal welfare regulations but also provided a tranquil environment for the animal inside each chamber. All chambers were equipped with an electronic automatic humidity cool steam injection system (1100-03239 HS-SINF Masalles, Barcelona, Spain) to ensure appropriate humidity in the inspirate (55 ± 10%). Ambient PO_2_, PCO_2_, humidity and temperature within each chamber were monitored via sensors, displayed and values recorded continuously via the Trends Building Management System of the University of Cambridge through a secure Redcare intranet. In this way, the percentage of oxygen in the isolators could be controlled with precision continuously over long periods of time, while minimizing maternal stress.^48^ Measurements of stress hormones in maternal plasma during chronic hypoxia confirmed lack of any change from baseline.^48^ This model of maternal chronic hypoxia also does not affect maternal food intake.^48^

At 105 d gestation, ewes were further split into 2 groups; receiving either a daily bolus (between 09:00 and 10:00) of intravenous Saline (0.6 mL/kg; Normoxic + Saline, NS, *n* = 8; Hypoxic + Saline, HS, *n* = 8) or Vitamin C dissolved in saline (200 mg/kg i.v. daily Ascorbate; A-5960; Sigma Chemicals, UK; 1.14 mmol/kg/day dissolved in 0.6 ml/kg saline; Normoxic + Vitamin C, NVC, *n* = 9; Hypoxic + Vitamin C, HVC, *n* = 9) from 105–135 d gestation. Vitamin C was chosen for administration in this study due to it being a commonly used water-soluble antioxidant supplement that can cross the placenta.^27,52^ We have previously established powerful antioxidant protection in the offspring by Vitamin C in several species, including the sheep.^12,27–30,53–55^ While Vitamin C can be easily administered both orally and daily in humans, the maternal i.v. route was chosen in sheep in this study to better control delivery into the maternal circulation. The rationale for a higher dose of Vitamin C used in this study versus that used in clinical trial (14.3 mg/kg/day^24^) was derived from previous studies in sheep pregnancy in our laboratory, which achieved elevations in circulating ascorbate concentrations within the required range for Vitamin C to compete effectively in vivo with nitric oxide in ovine pregnancy.^53,56,57^ To characterize effects in this model, maternal blood samples were taken at baseline (before infusion at 09:00–10:00 on 104–105 d gestation) and post treatment samples were taken 24 h after the first bolus at 106 d and then every 5 days thereafter (09:00–10:00) to measure plasma ascorbate concentration. Maternal Vitamin C treatment in both control and hypoxic ewes produced similar increments from baseline in maternal plasma Vitamin C, doubling and sustaining the circulating concentration over the period of treatment from 105 to 135 days of gestation.^27^ This dose effect is similar to the effect of Vitamin C treatment in human clinical trials, which also lead to a doubling of ascorbate concentrations in maternal plasma.^24^ We have previously demonstrated that this model of maternal chronic hypoxia^44^ and chronic Vitamin C exposure in normal pregnancy^45^ confers significant effects molecular regulation of fetal lung development.

### Post-mortem and sample collection

Fetuses were exposed to either maternal normoxia or chronic hypoxia from 105 d gestation until postmortem. Fetuses were evaluated near-term at 138 d gestation when the lung is in the alveolar stage of development similar to human late preterm birth (36–37 week of gestation; term = 40 weeks^50,51^). Immediately prior to euthanasia, a maternal blood sample was collected for analysis of plasma Vitamin C concentration. All ewes and their fetuses were euthanized by overdose of sodium pentobarbitone (0.4 mL/kg, slow intravenous administration, Pentoject; Animal Ltd, York, UK) and fetuses were delivered by hysterotomy. A fetal umbilical arterial blood sample was taken at post-mortem for measurement of plasma Vitamin C concentration. Fetal body and organ weights were recorded. A piece of left fetal lung tissue was snap frozen in liquid nitrogen and stored at −80°C for gene and protein expression analysis. A section of right fetal lung tissue was immersion fixed in 4% paraformaldehyde and processed to paraffin for immunohistochemical analysis.

The tissues generated in this study were part of a program of work designed with the primary objective of investigating cardiovascular physiology.^27^ This study used the tissues generated to address additional important scientific questions retrospectively, thereby making best use of the valuable experimental material. This scientific approach is strongly recommended by the UK Home Office 3 R principle of Replacement, Reduction and Refinement designed to ensure more humane animal research.^58^ Consequently, no developmental trajectory time points, lung tissue stereological analysis or prospective functional respiratory outcomes were performed.

### Maternal and fetal plasma Vitamin C analysis

Plasma concentrations of Vitamin C were measured by a fluorimetric technique using a centrifugal analyzer with a fluorescence attachment, according to the method of Vuilleumier and Keck,^59^ in collaboration with the Core Biochemical Assay Laboratory, Cambridge, UK as previously described.^27,45^

### Quantification of fetal lung mRNA expression

RNA was extracted and cDNA synthesized from fetal lung tissue samples ( ~50 mg; NS, *n* = 8; HS, *n* = 8; NVC, *n* = 9; HVC, *n* = 9) as previously described^45,60–62^ and following the MIQE guidelines.^63^ The expression of target genes regulating surfactant maturation, lung liquid secretion and reabsorption (regulated by chloride, sodium and water transport across the pulmonary epithelium), glucocorticoid signaling and hypoxia signaling^6,37,64^ were measured by quantitative reverse transcription polymerase chain reaction (qRT-PCR; Table 1), as previously described.^45,60–62,65,66^ The abundance of each transcript relative to the abundance of stable reference genes (beta-actin, peptidylprolyl isomerase, tyrosine 3-monooxygenase) was calculated using DataAssist 3.0 analysis software and is expressed as mRNA mean normalized expression (MNE) ± SD.^45,60–62^

**Table 1:** Target genes regulating surfactant maturation and lipid transport, fetal lung liquid movement, glucocorticoid signaling and hypoxia signaling by quantitative real-time RT-PCR (all primer sequences and concentrations previously published^44^).

	Gene Name	Protein Name	Function
Surfactant maturation and lipid transport			
Surfactant protein	S*FTP-A* S*FTP-B* S*FTP-C* S*FTP-D*	SP-A SP-B SP-C SP-D	Pulmonary immunity and surface tension regulating
Phosphate cytidylyltransferase 1, choline, alpha	*PCYT1A*	PCYT1A	Surfactant lipid synthesis
Fetal lung liquid movement			
Cystic fibrosis transmembrane conductance regulator	*CFTR*	CFTR	Chloride movement
Chloride channel voltage-sensitive 2 channel	*CLCN2*	CLCN2	Chloride movement
Epithelial sodium channel subunits	*SCNN1-A* *SCNN1-B* *SCNN1-G*	ENAC-α ENAC-β ENAC-γ	Sodium movement
Sodium potassium adenosine triphosphatase subunits	*ATP1-A1* *ATP1-B1*	Na-K-ATPase-α1 Na-K-ATPase-β1	Sodium movement
Aquaporins	*AQP-1* *AQP-3* *AQP-4* *AQP-5*	AQP-1 AQP-3 AQP-4 AQP-5	Water movement
Glucocorticoid signaling			
11β-hydroxysteroid dehydrogenase-1	*HSD11B-1*	11βHSD-1	Glucocorticoid activating enzyme isoform
11β-hydroxysteroid dehydrogenase-2	*HSD11B-2*	11βHSD-2	Glucorticoid de-activating enzyme isoform
Glucocorticoid receptor	*NR3C1*	GR	Cellular glucocorticoid receptor
NK2 homeobox 1 - thyroid transcription factor	*NKX2-1*	TTF-1	Glucorticoid responsive gene
Hypoxia signaling			
Hypoxia inducible factor subunits	*HIF-1α* *HIF-2α* *HIF-3α* *HIF-1b*	HIF-1α HIF-2α HIF-3α HIF-1β	Major regulators of hypoxia signalling
Egl-9 family hypoxia-inducible factor enzymes (encoding the prolyl hydroxylase domain proteins)	*EGLN-1* *EGLN-2* *EGLN-3*	PHD-2 PHD-1 PHD-3	Regulator of HIF activity and signaling
Vascular endothelial growth factor	*VEGF*	VEGF	Hypoxia responsive gene
Adrenomedullin	*ADM*	ADM	Hypoxia responsive gene
Lysine (K)-specific demethylase 3 A	*KDM3A*	JMJD1A	Hypoxia responsive gene
Solute carrier family 2, Facilitated glucose transporter member-1	*SLC2A1*	GLUT-1	Hypoxia responsive gene

### Quantification of fetal lung protein expression

Protein was extracted by sonication of fetal lung tissue ( ~100 mg, NS *n* = 6, NVC *n* = 7, HS *n* = 7, NVC *n* = 7;^45,61,67,68^). Protein content was determined by a MicroBCA Protein Assay Kit (PIERCE, Thermo Fisher Scientific Inc., Rockford, Illinois) and samples were then diluted to the same concentration so that a consistent volume is loaded into each well on the Western blot. Primary antibodies of interest were SP-B (1:1000, in 5% BSA in TBS-T, #WRAB-48604, Seven Hills Bioreagents; 8 kDa band); ENAC-β (1:1000, in 5% BSA in TBS-T, #PA5-77817, Invitrogen; 87 kDa band); Na^+^-K^+^-ATPase-α1 (1:1000 in 5% BSA in TBS-T, #MA3-929, Invitrogen; 110 kDa), Na^+^-K^+^-ATPase-β1 (1:1000 in 5% BSA in TBS-T, #MA3-930, Thermofisher; 50 kDa band); 11βHSD-2 (1:1000, in 5% BSA in TBS-T, #10004303, Cayman Chemical; 44 kDa band), GLUT-1 (1:1000, in 5% BSA in TBS-T, #CBL242, Chemicon Millipore; 46 kDa band). The primary antibodies were chosen based on genes that changed in response to antenatal Vitamin C administration in the fetal lung to determine if the transcriptional changes observed translated into protein abundance differences. Following incubation with the primary antibody, the blots were washed and incubated with the relevant species of Horse Radish Peroxidase labeled secondary IgG antibody for 1 h at room temperature. Enhanced chemiluminescence using SuperSignal West Pico Chemiluminescent Substrate (Thermo Scientific, Australia) was used to detect the blots. The Western blot was imaged using ImageQuant LAS4000 and the protein abundance was quantified by densitometry using Image quant software (GE Healthcare, Victoria, Australia). Total target protein abundance was then normalized to total protein (Ponceau S) or to a reference protein, β-actin (1:10,000 in 5% BSA in TBS-T, ATCB HRP conjugate, #4967, Cell Signaling Technology; 42 kDa band), β-tubulin (1:10,000 in 5% BSA in TBS-T, Beta TUBULIN (9F3) HRP conjugate, #5346, Cell Signaling Technology; 55 kDa band) or COX1V (3E11) (1:10,000 in 5% BSA in TBS-T, COX1V (3E11) HRP conjugate, #5247 P, Cell Signaling Technology; 17 kDa band).

### Quantification of surfactant producing cells within lung tissue

To determine the effect of chronic fetal hypoxia and antenatal Vitamin C administration on the surfactant-producing capacity of the lung at the structural level, immunohistochemistry was performed (NS = 8; NVC = 9; HS = 8; HVC = 9) using a rabbit anti-human mature surfactant protein B (SP-B) antibody (1:500, WRAB-48604, Seven Hills Bioreagents, Ohio), as previously described.^44,45^ Sections were examined using Visiopharm new Computer Assisted Stereological Toolbox (NewCAST) software (Visiopharm, Hoersholm, Denmark) and point counting was used to determine the numerical density of SP-B positive cells present in the alveolar epithelium of lung tissue, as previously described.^44,45,69^

### Statistical analyses

All statistical analyses were performed using GraphPad Prism (v8). All data were evaluated for outliers ± 2 SD from the mean for each treatment group (only 1 round of outlier removal was performed). Data in figures and table are presented as Log2 fold change of mean normalized expression ± SD to demonstrate the effect of Vitamin C compared to respective saline Control group for both Normoxic (*n* = 9; NVC vs mean of NS group) and Hypoxic (*n* = 9; HVC vs mean of HS group) fetuses.

All data were checked for normality and if normally distributed data were compared using the Student’s *t*-test for unpaired data and if not normally distributed data were compared using the Mann-Whitney test. Statistical analysis was undertaken firstly to determine if there was a significant effect of Vitamin C within each treatment group (i.e. NVC vs NS group and HVC vs HS group) and secondly to determine if there was a significant difference between the effect of antenatal Vitamin C between Normoxic and Hypoxic fetuses. For all comparisons, *P* < 0.05 was considered statistically significant.

## Results

### Plasma Vitamin C and fetal growth

**Fig. 1:**
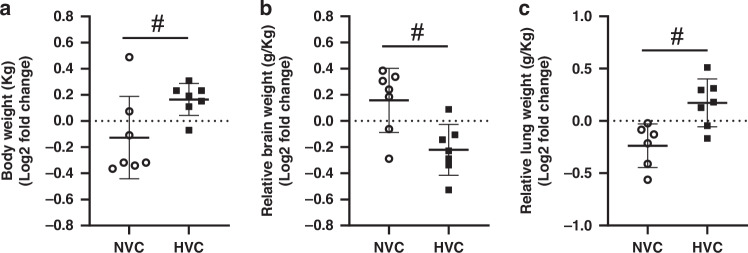
Effect of maternal Vitamin C on fetal growth. Fetal body weight **a**, relative brain weight **b** and relative lung weight **c**. Data presented as Log2 fold change (compared to respective saline group) mean ± SD in the Normoxic Vitamin C (NVC; white circles, *n* = 9) and Hypoxic Vitamin C (HVC; black squares; *n* = 9) fetal lung. All data were evaluated for outliers ± 2 SD from the mean for each treatment group. Positive or negative mean values indicate induction or repression of gene expression, respectively. #*P* < 0.05; significant difference between Vitamin C effectiveness in the normally grown vs. growth restricted fetal lung (i.e. NVC vs. HVC).

Maternal plasma Vitamin C levels did not differ between groups at baseline (105 d gestation; N: 38.0 ± 3.9 µmol/L; H: 36.2 ± 3.1 µmol/L; HVC: 44.1 ± 4.5 µmol/L; NVC: 43.8 ± 3.3 µmol/L). Maternal treatment with Vitamin C significantly increased plasma Vitamin C concentration to similar levels in samples taken just prior to post-mortem in the treated Normoxic (81.4 ± 12.8 µmol/L) and Hypoxic pregnant ewes (71.6 ± 6.0 µmol/L), approximately doubling basal levels (*P* < 0.05). Plasma levels of Vitamin C measured in the sample taken from the fetal umbilical artery at post-mortem were also similarly elevated in fetuses from Normoxic and Hypoxic ewes treated with Vitamin C compared to those from pregnancies treated with saline alone (N: 20.2 ± 1.2 µmol/L; H: 22.3 ± 3.8 µmol/L; NVC: 30.1 ± 1.4 µmol/L; HVC: 33.1 ± 1.2 µmol/L; *P* < 0.05). There was no significant effect of antenatal Vitamin C on fetal body, relative brain weight or relative lung weight in both normally grown and growth restricted fetuses (Fig. 1a–c). However, there was, a significant effect of exposure to maternal chronic hypoxia, which is driven by the induction of asymmetric fetal growth restriction compared to normally growth fetuses (Fig. 1a–c).

### Regulation of surfactant maturation

**Fig. 2:**
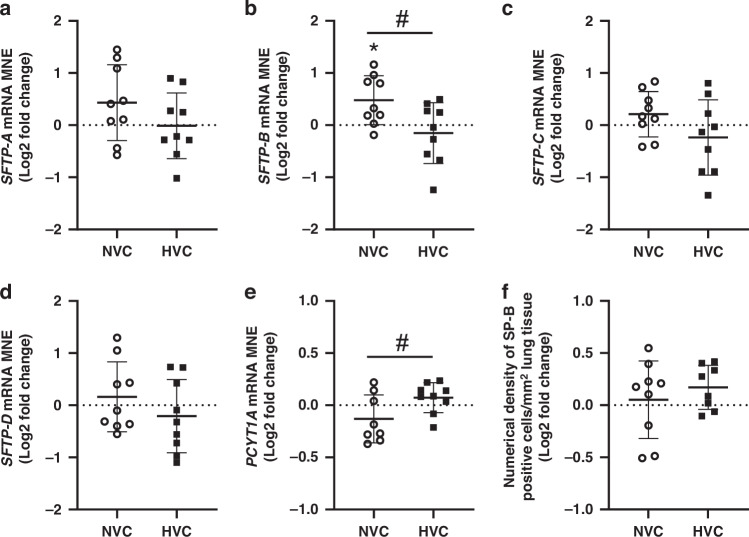
Effect of maternal Vitamin C on expression of surfactant maturation in lung of normally grown and growth restricted fetuses. Mean normalized gene expression (MNE) of surfactant protein (*SFTP*)*-A* **a**, *-B* **b**, *-C* **c**, *-D* **d** and phosphate cytidylyl transferase 1, choline, alpha (*PCYT1A*; **e**). Surfactant protein-producing capacity of the lung was determined by quantitating the numerical density of SP-B positive cells in the alveolar epithelium of lung tissue **f**. Data presented as Log2 fold change (compared to respective saline group) mean ± SD in the Normoxic Vitamin C (NVC; white circles, *n* = 9) and Hypoxic Vitamin C (HVC; black squares; *n* = 9) fetal lung. All data were evaluated for outliers ± 2 SD from the mean for each treatment group. Positive or negative mean values indicate induction or repression of gene expression, respectively. *P < 0.05; compared to respective control group, i.e. N vs. NVC or H vs. HVC. #*P* < 0.05; significant difference between Vitamin C effectiveness in the normally grown vs growth-restricted fetal lung (i.e NVC vs. HVC).

There was a significant effect of antenatal Vitamin C treatment increasing *SFTP-B* mRNA expression in the lung of Normoxic but not Hypoxic fetuses (Fig. 2b). There was a differential effect of antenatal Vitamin C treatment between Normoxic and Hypoxic fetuses, with increased expression of *SFTP-B* (Fig. 2b) and decreased surfactant phospholipid marker *PCYT1A* (Fig. 2e) in the lung of Normoxic compared to Hypoxic fetuses. There was no significant effect of antenatal Vitamin C treatment on expression of *SFTP-A*, *SFTP-C* and *SFTP-D* or numerical density of SP-B positive cells in the alveolar epithelium between Normoxic and Hypoxic fetuses (Fig. 2a, c, d, f).

### Expression of genes regulating fetal lung liquid movement

**Fig. 3:**
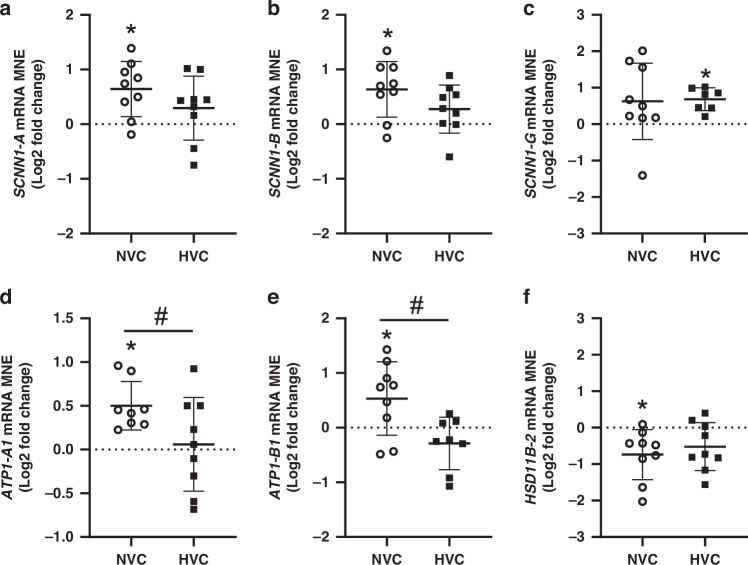
Effect of maternal Vitamin C on expression of genes regulating sodium movement and glucocorticoid availability in the normally grown and growth-restricted fetal lung. Epithelial sodium channel (*SCNN1*)*-A* **a**, *-B* **b** and *-G* **c** subunits, sodium potassium ATPase-A1 (*ATP1-A1*, **d**) sodium potassium ATPase-B1 (*ATP1-B1*, **e**) and glucocorticoid activating enzyme *HSD11B-2* **f**. Gene data presented as Log2 fold change (compared to respective saline group) mean normalized expression (MNE) ± SD in the Normoxic Vitamin C (NVC; white circles, *n* = 9) and Hypoxic Vitamin C (HVC; black squares; *n* = 9) fetal lung. All data were evaluated for outliers ± 2 SD from the mean for each treatment group. Positive or negative mean values indicate induction or repression of gene expression, respectively. *P < 0.05; compared to respective control group, i.e. N vs. NVC or H vs. HVC. #*P* < 0.05; significant difference between Vitamin C effectiveness in the normally grown vs growth-restricted fetal lung (i.e NVC vs. HVC).

There was a significant effect of antenatal Vitamin C treatment increasing *SCNN1-A*, *SCNN1-B*, *ATP1-A1* and *ATP1-B1* in the lung of Normoxic but not Hypoxic fetuses (Fig. 3a, b, d, e). Conversely, antenatal Vitamin C treatment significantly increased expression of *SCNN1-G* in the lung of Hypoxic, but not Normoxic fetuses (Fig. 3c). There was a differential effect of antenatal Vitamin C treatment on expression of *ATP1-A1* and *ATP1-B1* between Normoxic and Hypoxic fetuses (Fig. 3d, e). In contrast, there was no effect of antenatal Vitamin C treatment on the expression of genes regulating chloride movement (*CFTR* or *CLCN2)* or water movement (*AQP-1*, *AQP-3*, *AQP-4* or *AQP-5)* in the lung of Normoxic and Hypoxic fetuses (Table 2).

**Table 2:** Effect of antenatal Vitamin C treatment on expression of genes regulating fetal lung development in normoxic and hypoxic fetal sheep

Gene family	Gene symbol	NVC	HVC
Fetal lung liquid movement	*CFTR*	−0.09 ± 0.38	−0.18 ± 0.35
	*CLCN2*	0.05 ± 0.30	−0.04 ± 0.27
	*AQP-1*	0.09 ± 0.28	−0.18 ± 0.38
	*AQP-3*	−0.01 ± 0.49	0.10 ± 0.91
	*AQP-4*	0.23 ± 0.67	−0.64 ± 1.13
	*AQP-5*	0.22 ± 0.48	0.16 ± 0.53
Glucocorticoid signaling	*HSD11B-1*	0.11 ± 0.19	−0.03 ± 0.61
	*NR3C1*	0.16 ± 0.21	0.33 ± 0.16
	*NR3C2*	0.06 ± 0.61	0.01 ± 0.24
	*NKX2-1*	0.24 ± 0.33	0.22 ± 0.46
Hypoxia signaling	*HIF-1A*	0.12 ± 0.33	0.15 ± 0.21
	*HIF-1B*	0.126 ± 0.278	0.129 ± 0.366
	*EGLN-1*	−0.002 ± 0.245	0.028 ± 0.331
	*EGLN-2*	−0.009 ± 0.268	0.077 ± 0.213
	*KDM3A*	0.14 ± 0.24	−0.04 ± 0.23

Data are presented as mean Log2 fold change (i.e. NVC/HVC compared to mean of respective saline group) mean normalized gene expression ± SD. Positive or negative mean values indicate induction or repression of gene expression, respectively. *P* < 0.05 was considered statistically significant.

### Expression of genes regulating glucocorticoid signaling

There was a significant effect of antenatal Vitamin C treatment decreasing the mRNA expression of glucocorticoid deactivating enzyme *HSD11β-2* in the lung of Normoxic but not Hypoxic fetuses (Fig. 3f). In contrast, there was no significant effect of antenatal Vitamin C on the mRNA expression of the glucocorticoid activating enzyme *HSD11β-1* or the receptors for downstream glucocorticoid signaling (*NR3C1* and *NR3C2*) or on the glucocorticoid responsive gene *NKX2-1* in the lung of Normoxic or Hypoxic fetuses or the magnitude of effect between them (Table 2).

### Expression of genes regulating hypoxia signaling

**Fig. 4:**
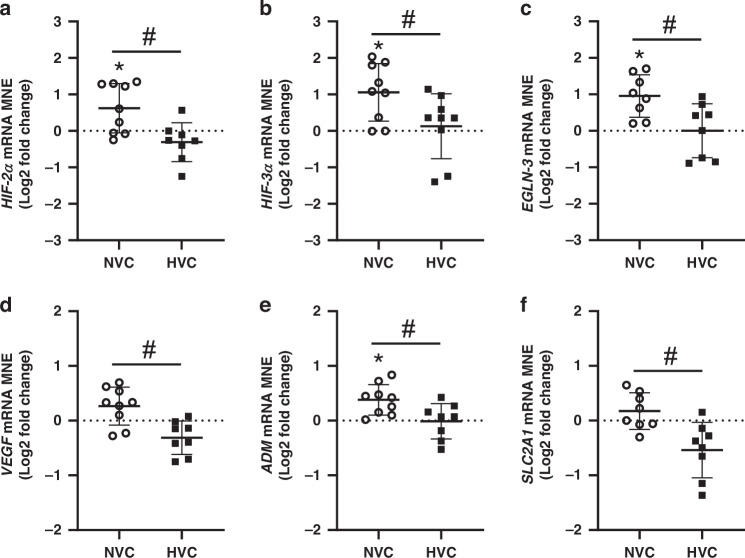
Effect of maternal Vitamin C on expression of genes regulating hypoxia signaling in the normally grown and growth restricted fetal lung. Hypoxia inducible factor (*HIF*)*-2α* **a** and *-3α* **b** subunits, prolyl hydroxylase domain (*EGLN*)*-3* **c** and hypoxia-responsive genes vascular endothelial growth factor (*VEGF*, **d**); adrenomedullin (*ADM*, **e**) and facilitated glucose transporter 1 (*SLC2A1*, **f**). Gene data presented as Log2 fold change (compared to respective saline group) mean normalized expression (MNE) ± SD in the Normoxic Vitamin C (NVC; white circles, *n* = 9) and Hypoxic Vitamin C (HVC; black squares; *n* = 9) fetal lung. All data were evaluated for outliers ± 2 SD from the mean for each treatment group. Positive or negative mean values indicate induction or repression of gene expression, respectively. *P < 0.05; compared to respective control group, i.e. N vs. NVC or H vs. HVC. #*P* < 0.05; significant difference between Vitamin C effectiveness in the normally grown vs growth-restricted fetal lung (i.e NVC vs. HVC).

There was a significant effect of antenatal Vitamin C treatment upregulating the expression of genes regulating hypoxia signaling (*HIF-2α*, *HIF-3α*, *EGLN-3*) and the hypoxia responsive gene *ADM* in the lung of Normoxic but not Hypoxic fetuses (Fig. 4a–c, e). There was a significant differential response to antenatal Vitamin C in the lung expression of *HIF-2α*, *HIF-3α*, *EGLN-3, VEGF, ADM* and *SLC2A1* between Normoxic and Hypoxic fetuses (Fig. 4). Conversely, there was no significant effect of antenatal Vitamin C or exposure to Hypoxia on expression of *HIF-1α*, *HIF-1β, EGLN-1*, *EGLN-2* or *KDM3A* (Table 2).

### Protein expression of genes in lung signaling pathways that changed in response to antenatal Vitamin C treatment

**Fig. 5:**
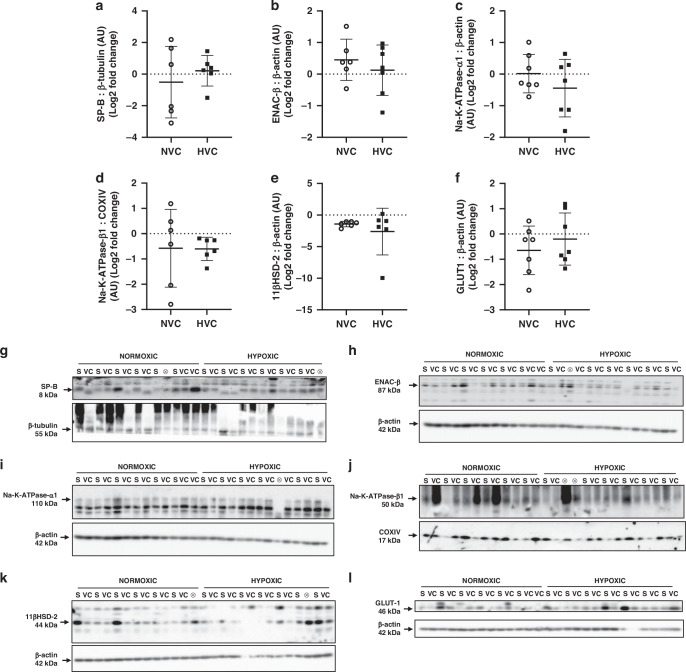
Protein expression for genes that changed in the fetal lung in response to antenatal Vitamin C administration. Data presented as normalized protein expression in arbitrary units (AU) for SP-B (**a**; 8 kDa band), ENAC-ß (**b**; 87 kDa band), Na-K-ATPase-α1 (**c**; 110 kDa band), Na-K-ATPase-ß1 (**d**; 50 kDa band), 11ßHSD-2 (**e**; 44 kDa band), GLUT-1 (**f**; 46 kDa band). Western blot images represent target protein (upper panel) and reference protein (lower panel) for fetuses in normoxic + saline (NS), normoxic + Vitamin C (NVC), hypoxic + saline (HS) and hypoxic + Vitamin C (HVC) fetuses. ⊗ = Outliers (all data were evaluated for outliers ± 2 standard deviations from mean of each treatment group) not included in analysis. Beta actin (ß-actin; **h**, **i**, **k**, **l**; 42 kDa band), Cytochrome oxidase IV (COXIV; **j**; 17 kDa band), and beta-tubulin (ß-tubulin; **g**; 55 kDa band) are obtained from the same gel. Positive or negative mean values indicate induction or repression of gene expression, respectively. *P* < 0.05 was considered statistically significant.

To investigate if there were effects on translation potential in the fetal lung, we investigated the protein expression of a panel of genes from signaling pathways that changed in response to antenatal Vitamin C exposure. There was no significant effect of antenatal Vitamin C treatment or hypoxia on the lung protein expression of SP-B, factors regulating sodium movement (ENAC-ß, Na-K-ATPase-α1, Na-K-ATPase-ß1), glucocorticoid activity (11ßHSD-2) or on the hypoxia-responsive factor GLUT-1 (Fig. 5).

## Discussion

The data show a differential effect of maternal Vitamin C treatment on regulation of genes involved in surfactant maturation, sodium movement and hypoxia signaling between the normoxic normally grown sheep fetus and the chronically hypoxic growth restricted sheep fetus. We demonstrate variability in the magnitude, and in some cases the direction, of the effects of maternal treatment with Vitamin C on the gene expression of pathways involved in lung development between the Normoxic and Hypoxic sheep fetus.

In normoxic fetal sheep, maternal treatment with Vitamin C during late gestation significantly increases the lung gene expression of factors regulating surfactant production, sodium transport (key regulator of lung liquid movement in fetal life), glucocorticoid availability and hypoxia signaling^45^ (see summary in Table 3). Here, we show that maternal treatment with Vitamin C in the hypoxic growth restricted sheep fetus compared to the normoxic sheep fetus has very limited effects in the magnitude (except for an increase in *SCNN1-G* expression) and, in some cases, differential effects in the direction of changes in gene expression pathways involved in lung development compared to the normally grown fetus (see summary in Table 3). Using this model of maternal chronic hypoxia for a month in late gestation to induce fetal growth restriction,^27^ we have also reported that the chronically hypoxic sheep fetus compared to the normoxic sheep fetus in untreated pregnancies shows increased expression of molecular markers regulating surfactant maturation and lipid transport, lung liquid movement (markers of sodium and water movement) and hypoxia signaling in the fetal lung^44^ (see summary in Table 3). Therefore, rather than signifying resistance to the antenatal Vitamin C treatment, the most likely explanation for a lack or very limited effect of maternal treatment with Vitamin C in the hypoxic fetus in this study is that molecular signaling pathways in the lung are maximally upregulated in response to the chronic hypoxic insult in this model; thus, added exposure to Vitamin C is unable to upregulate the system further. Indeed, this hypothesis is supported by the findings from this study and our previous studies whereby molecular marker *SFTP-B* expression was increased by 53% in response to maternal chronic hypoxia (0.85 ± 0.22 NS vs. 1.30 ± 0.44 HS MNE; *P* = 0.02) and increased 46% in response to antenatal Vitamin C in normoxic pregnancy (0.85 ± 0.22 NS vs. 1.24 ± 0.40 NVC MNE; *P* = 0.03). However, in the lung of the chronically hypoxic fetus exposed to Vitamin C, there was a similar increase from Control values (47%; 0.85 ± 0.22 NS vs. 1.25 ± 0.46 HVC MNE; *P* = 0.04), but when compared to the lung exposure to maternal chronic hypoxia alone there was only a 4% difference (1.30 ± 0.44 HS vs 1.25 ± 0.45 HVC MNE; *P* = 0.83). Hence this study provides evidence that there is no synergistic upregulation of surfactant markers in the lung of fetuses exposed to both maternal chronic hypoxia and antenatal Vitamin C, supporting a maximal upregulation of the system in response to the chronic hypoxia insult alone. In addition, this study highlights that while antenatal Vitamin C exposure has differential effects on molecular regulation of the lung in both normally grown and growth-restricted fetuses, in this cohort there was no effect of Vitamin C exposure on fetal body, relative lung weight, or the number of SP-B producing cells in the alveolar epithelium of fetal lung tissue.

**Table 3:** Summary of gene expression outcome of studies using this fetal sheep model to investigate the effect of maternal chronic hypoxia and antenatal Vitamin C exposure on molecular regulation of fetal lung maturation.

This table includes summary of data included in 2 previously published studies; (1) Normoxic (N) vs Hypoxic (H) fetal lambs^44^ and (2) Normoxic Saline (NS) vs Normoxic Vitamin C (NVC) fetal lambs^45^. The findings of the current study build on this to include (1) the effect on molecular markers in the lung of H vs Hypoxic Vitamin C (HVC) lambs and (2) the magnitude of responsiveness to Vitamin C in the lung of normoxic (NVC) and hypoxic (HVC) fetuses. ↑↓=*P < 0.05; compared to respective control group in column 1 (N v H), column 2 (N v NVC) or column 3 (H v HVC) or Vitamin C effectiveness in the normally grown vs growth restricted fetal lung (column 4 = NVC vs. HVC fold change relative to respective control). ↔ no significant difference between groups. For full description of gene markers and their roles see Table 1.

The analysis in this study highlights a significant difference between responsiveness of the normally grown and growth-restricted fetal lung to antenatal Vitamin C treatment in key molecular developmental pathways. There was a differential response to Vitamin C with higher gene expression of *SFTP-B*, *ATP1A1*, *ATP1B1*, *HIF-2α*, *HIF-3α*, *EGLN-3*, *VEGF*, *ADM*, *SLC2A1* and lower gene expression of *PCYT1A* in the lung of the normally grown fetus compared with the growth restricted fetal lung. It is clear that exposure to a single insult in this sheep model, either maternal chronic hypoxia^44^ or antenatal Vitamin C administration in healthy pregnancy,^45^ can promote upregulation of genes regulating fetal lung development, but synergistic exposure to both insults did not confer further benefit (see summary in Table 3). We have previously shown benefits of maternal chronic hypoxia (decreasing expression of prooxidant maker *NOX-4* and increasing antioxidant marker *CAT*^44^) and antenatal Vitamin C treatment in normoxic pregnancy (increasing expression of antioxidant marker *SOD-1*^45^). However, due to lack of sample availability, in this study we were unable to investigate the impact of exposure to maternal chronic hypoxia and antenatal Vitamin C treatment on molecular regulation of oxidative stress in the fetal lung. While there were significant effects of either exposure to antenatal chronic hypoxia or Vitamin C on gene expression in the fetal lung in this study, there were no differences in the expression of a panel of proteins involved in surfactant maturation, sodium movement, glucocorticoid availability, and hypoxia-responsive proteins. Therefore, the increase in gene expression may serve as a local reservoir of molecular regulators that can be drawn upon in response to developmental challenges, such as acute hypoxic challenge or preterm exposure to the air-breathing environment at birth. Conversely, while beyond the scope of this study, there may be epigenetic posttranscriptional changes programmed by exposure to maternal chronic hypoxia on translation of genes in these signaling pathways. Future studies generating new tissue cohorts are required to specifically investigate these mechanisms.

Table 3 provides a direct comparison between studies investigating gene expression markers of fetal lung development using this sheep model of exposure to maternal chronic hypoxia and/or antenatal Vitamin C. Of interest, there is considerable overlap in the expression of genes that are upregulated in the fetal lung by Vitamin C treatment in normoxic pregnancy (10 genes; those regulating hypoxia signaling and feedback = *HIF-2α*, *HIF-3α*, *EGLN-3, ADM*; glucocorticoid availability = *HSD11B-2*; sodium movement = *SCNN1-A*, *SCNN1-B*, *ATP1-A1*, *ATP1-B1;* and surfactant maturation = *SFTP-B*^45^) and in the expression of genes that are upregulated in the fetal lung by exposure to chronic hypoxia alone in untreated pregnancy (10 genes; those regulating hypoxia signaling and feedback = *HIF-3α*, *EGLN-3, KDM3A*, *SLC2A1*; sodium movement = *SCNN1-B*, *ATP1-A1*, *ATP1-B1*; water movement = *AQP4*; and surfactant maturation = *SFTP-B*, *SFTP-D*^44^). However, there are several changes within these signaling pathways that are unique to either exposure to chronic hypoxia in untreated pregnancy or treatment with Vitamin C in normoxic pregnancy. Therefore, exposure of the fetal lung to different stimuli in normoxic and hypoxic fetal sheep can generate individual molecular signatures regulating fetal lung maturation.

While there is limited understanding of the effect of antenatal Vitamin C on the fetal lung, there is further evidence of the complex direct and indirect regulatory role and interaction between growth restriction, oxidative stress and antenatal antioxidants in postnatal life. In a sheep model of growth restriction induced by single uterine umbilical artery ligation at 105 d gestation, chronically hypoxic growth-restricted lambs (at 24 h of age) had altered structural lung development but daily antioxidant melatonin treatment did not protect against this.^70^ In a further study, antenatal melatonin treatment for the last third of gestation modulated pulmonary responsiveness, antioxidant capacity and prooxidant status in the lung of chronically hypoxic fetal sheep at 12 days after birth.^40^ These studies provide insights into the differential effects of growth restriction models on the postnatal lung and considerations for the role of antenatal antioxidants in developmental programming of responsiveness to hypoxic/hyperoxic challenges in postnatal life. Indeed, at the cellular level, a differential effect of chronic or intermittent hypoxia-hyperoxia on mitochondrial structure and function in fetal airway smooth muscle cells highlights the importance of physiological balance between overloading the system with antioxidants and removing reactive oxygen species below physiological levels.^71^

There is a critical need to investigate candidate antenatal interventions extensively in pre-clinical studies taking into account obstetric subpopulations prior to therapeutic use clinically to avoid unintended consequences on the fetus and organ systems as a result of heterogeneous causes of pregnancy complications.^5,12,72–74^ In a model of intrauterine inflammation in a premature mouse model, antenatal melatonin normalized markers of inflammation, SP-B expression in the fetal lung (E18) and lung structure in the neonatal lung (d1) and showed visual differences in magnitude of effect between Control and mice with intrauterine inflammation induced by lipopolysaccharide exposure.^75^ When investigating antenatal therapeutics, it is also important to consider interaction with other commonly administered antenatal treatments, for example exposure to antenatal glucocorticoids in extremely preterm newborns has some antioxidant effects, reducing postnatal oxidative stress and endogenous antioxidant activity with a greater effect observed in females.^76^

While our findings provide further mechanistic insights into the role of antenatal Vitamin C on regulation of lung development of the normally grown and growth restricted sheep fetus, there are some important limitations of this study. First, while we controlled for as many factors as possible (including, pen floor surface area, humidity, temperature, noise levels, feeding regimen, remote monitoring systems, mitigating exposure to excessive noise and visibility of other sheep to reduce stress), Control ewes were housed in floor pens while the animals exposed to hypoxia were housed in the bespoke chambers. However, we believe this did not have a significant effect on maternal stress between groups, evidenced by no change from baseline in maternal stress hormones or food intake.^48^

Secondly, while the dose of Vitamin C administered to ewes in this model to achieve antioxidant effects in the fetus is significantly higher than the dose used in human clinical trials, the relative dose effect resulting in doubling of maternal plasma ascorbate concentrations is consistent with human clinical studies.^24,27,53^ While the values achieved for ascorbate concentrations are lower in the fetus than in the mother, little is known about the transport of Vitamin C in the ovine placenta or the factors affecting it. This is an area worthy of deeper investigation and consideration towards clinically applicable and translatable outcomes. Particularly as when investigating interventions to overcome the effect of oxidative stress in pregnancies complicated by growth restriction, poor placental development and/or function will likely contribute to heterogeneity in transport and therefore impact on responsiveness at the organ level.

Thirdly, as the tissues generated were part of a program of work designed with the primary objective of investigating cardiovascular physiology,^27,48^ only molecular studies were able to be undertaken and as such we do not know the lung structural and functional effects on the transition to air-breathing or lung function. As such we are unable to compare fetal lung function outcomes after antenatal Vitamin C exposure with the existing clinical studies during infancy. Fourth, only male fetuses were used in this study (females were assigned to a postnatal study) so we are unable to interrogate potential sex differences. Finally, this study has focused on characterizing antenatal Vitamin C exposure in one model of fetal growth restriction induced by maternal isobaric hypoxia and it is well documented that effects of chronic hypoxemia are regulated by the timing, severity and duration of the insult.^5,6,10^ From a mechanistic understanding, it would be interesting to investigate if the effect of antenatal Vitamin C exposure would have a more significant effect at the molecular level in the fetal lung in a model of early growth restriction such as placental restriction induced by uterine carunclectomy or single uterine artery ligation, which in itself downregulates molecular markers of lung maturation.^6,10^ Such studies would help elucidate further if the capacity for Vitamin C to maximally upregulate molecular signaling in the fetal lung is different between hypoxic fetuses produced by different types of adverse intrauterine conditions.

In summary, the data presented in this study provide molecular insights into the heterogeneity of the lung’s response to antenatal Vitamin C treatment in fetuses from normal pregnancies and those from pregnancies complicated by chronic hypoxia and fetal growth restriction. Specifically, the effectiveness of antenatal intervention with Vitamin C in complicated pregnancy may depend on the timing, severity and duration of the suboptimal gestational condition. Variable molecular responses to antenatal interventions in normally grown and growth restricted fetuses highlight the need for further investigation of antenatal interventions in preclinical animal models of fetuses of compromised pregnancy prior to human use to ensure benefits of effective therapeutic intervention outweighs potential harm.

## Data avaliability

All data supporting the results are presented in the manuscript.

## References

[1] Kumar, V. H. & Ryan, R. M. Growth factors in the fetal and neonatal lung. *Front. Biosci.* **9**, 464–480 (2004). 14766383 10.2741/1245

[2] Flecknoe, S., Harding, R., Maritz, G. & Hooper, S. Increased lung expansion alters the proportions of type I and type II alveolar epithelial cells in fetal sheep. *Am. J. Physiol.-Lung Cell. Mol. Physiol.* **278**, L1180–L1185 (2000). 10835323 10.1152/ajplung.2000.278.6.L1180

[3] Hooper, S. & Harding, R. Fetal lung liquid: a major determinant of the growth and functional development of the fetal lung. *Clin. Exp. Pharmacol. Physiol.* **22**, 235–241 (1995). 7671435 10.1111/j.1440-1681.1995.tb01988.x

[4] Fowden, A. & Forhead, A. Endocrine mechanisms of intrauterine programming. *Reproduction* **127**, 515–526 (2004). 15129007 10.1530/rep.1.00033

[5] Morrison, J. L. Sheep models of intrauterine growth restriction: fetal adaptations and consequences. *Clin. Exp. Pharmacol. Physiol.* **35**, 730–743 (2008). 18498533 10.1111/j.1440-1681.2008.04975.x

[6] McGillick, E. V., Orgeig, S., Giussani, D. A. & Morrison, J. L. Chronic hypoxaemia as a molecular regulator of fetal lung development: implications for risk of respiratory complications at birth. *Paediatr. Respir. Rev.* **S1526-0542**, 30085–30089 (2016). 10.1016/j.prrv.2016.08.011 27692868

[7] McGillick, E. V., Orgeig, S., Williams, M. T. & Morrison, J. L. Risk of respiratory distress syndrome and efficacy of glucocorticoids: are they the same in the normally grown and growth restricted infant? *Reprod. Sci.* **23**, 1459–1472 (2016). 27549917 10.1177/1933719116660842

[8] Robert, M., Neff, R., Hubbell, J., Taeusch, H. & Avery, M. Association between maternal diabetes and the respiratory-distress syndrome in the newborn. *N. Engl. J. Med.* **294**, 357–360 (1976). 1246288 10.1056/NEJM197602122940702

[9] McGillick, E. V., Lock, M. C., Orgeig, S. & Morrison, J. L. Maternal Obesity Mediated Predisposition to Respiratory Complications at Birth and in Later Life: Understanding the Implications of the Obesogenic Intrauterine Environment. *Paediatric Respiratory Reviews*, (2016). 10.1016/j.prrv.2016.10.003 27818069

[10] Darby, J. R., Varcoe, T. J., Orgeig, S. & Morrison, J. L. Cardiorespiratory consequences of intrauterine growth restriction: influence of timing, severity and duration of hypoxaemia. *Theriogenology* **150**, 84–95 (2020). 32088029 10.1016/j.theriogenology.2020.01.080

[11] McMillen, I. C. & Robinson, J. S. Developmental origins of the metabolic syndrome: prediction, plasticity, and programming. *Physio. Rev.* **85**, 571–633 (2005). 10.1152/physrev.00053.2003 15788706

[12] Giussani, D. A. Breath of life: heart disease link to developmental hypoxia. *Circulation* **144**, 1429–1443 (2021). 34694887 10.1161/CIRCULATIONAHA.121.054689 PMC8542082

[13] Barker, D. J., Osmond, C., Golding, J., Kuh, D. & Wadsworth, M. Growth in utero, blood pressure in childhood and adult life, and mortality from cardiovascular disease. *Br. Med. J.* **298**, 564–567 (1989). 2495113 10.1136/bmj.298.6673.564 PMC1835925

[14] Walther, F. J., Wade, A. B., Warburton, D. & Forman, H. J. Ontogeny of antioxidant enzymes in the fetal lamb lung. *Exp. Lung Res.* **17**, 39–45 (1991). 2013272 10.3109/01902149109063280

[15] Georgeson, G. D. et al. Antioxidant enzyme activities are decreased in preterm infants and in neonates born via caesarean section. *Eur. J. Obstet. Gynecol. Reprod. Biol.* **103**, 136–139 (2002). 12069735 10.1016/s0301-2115(02)00050-7

[16] Asikainen, T. M., Raivio, K. O., Saksela, M. & Kinnula, V. L. Expression and developmental profile of antioxidant enzymes in human lung and liver. *Am. J. Respiratory Cell Mol. Biol.* **19**, 942–949 (1998). 10.1165/ajrcmb.19.6.3248 9843929

[17] Frank, L. & Groseclose, E. E. Preparation for birth into an O2-rich environment: the antioxidant enzymes in the developing rabbit lung. *Pediatr. Res.* **18**, 240–244 (1984). 6728556 10.1203/00006450-198403000-00004

[18] Al-Gubory, K. H., Fowler, P. A. & Garrel, C. The roles of cellular reactive oxygen species, oxidative stress and antioxidants in pregnancy outcomes. *Int. J. Biochem. Cell Biol.* **42**, 1634–1650 (2010). 20601089 10.1016/j.biocel.2010.06.001

[19] Davis, J. M. & Auten, R. L. Maturation of the antioxidant system and the effects on preterm birth. *Semin. Fetal Neonatal Med.* **15**, 191–195 (2010). 20452845 10.1016/j.siny.2010.04.001

[20] Loverro, G. et al. Lipoperoxidation and antioxidant enzymes activity in pregnancy complicated with hypertension. *Eur. J. Obstet. Gynecol. Reprod. Biol.* **70**, 123–127 (1996). 9119090 10.1016/s0301-2115(95)02561-8

[21] Poranena, A.-K., Ekblad, U., Uotila, P. & Ahotupa, M. Lipid peroxidation and antioxidants in normal and pre-eclamptic pregnancies. *Placenta* **17**, 401–405 (1996). 8899868 10.1016/s0143-4004(96)90021-1

[22] Ilhan, N., Celik, E. & Kumbak, B. Maternal plasma levels of interleukin-6, C-reactive protein, vitamins C, E and A, 8-isoprostane and oxidative status in women with preterm premature rupture of membranes. *J. Matern.-Fetal Neonatal Med.* **28**, 316–319 (2015). 24749795 10.3109/14767058.2014.916674

[23] Wideman, G. L., Baird, G. H. & Bolding, O. T. Ascorbic acid deficiency and premature rupture of fetal membranes. *Am. J. Obstet. Gynecol.* **88**, 592–595 (1964). 14128191 10.1016/0002-9378(64)90885-3

[24] Poston, L. et al. Vitamin C and vitamin E in pregnant women at risk for pre-eclampsia (vip trial): randomised placebo-controlled trial. *Lancet* **367**, 1145–1154 (2006). 16616557 10.1016/S0140-6736(06)68433-X

[25] Chappell, L. C. et al. Vitamin C and E supplementation in women at risk of preeclampsia is associated with changes in indices of oxidative stress and placental function. *Am. J. Obstet. Gynecol.* **187**, 777–784 (2002). 12237663 10.1067/mob.2002.125735

[26] Greenough, A., Shaheen, S. O., Shennan, A., Seed, P. T. & Poston, L. Respiratory outcomes in early childhood following antenatal vitamin C and E Supplementation. *Thorax* **65**, 998–1003 (2010). 20889523 10.1136/thx.2010.139915

[27] Brain, K. et al. Intervention against Hypertension In The Next Generation Programmed By Developmental Hypoxia. *PLoS Biol.* **17**, e2006552 (2019). 30668572 10.1371/journal.pbio.2006552 PMC6342530

[28] Kane, A. D., Herrera, E. A., Camm, E. J. & Giussani, D. A. Vitamin C prevents intrauterine programming of in vivo cardiovascular dysfunction in the rat. *Circ. J.* **77**, 2604–2611 (2013). 23856654 10.1253/circj.cj-13-0311

[29] Giussani, D. A. et al. Developmental programming of cardiovascular dysfunction by prenatal hypoxia and oxidative stress. *PLoS One* **7**, e31017 (2012). 22348036 10.1371/journal.pone.0031017 PMC3278440

[30] Richter, H. et al. Ascorbate prevents placental oxidative stress and enhances birth weight in hypoxic pregnancy in rats. *J. Physiol.* **590**, 1377–1387 (2012). 22289909 10.1113/jphysiol.2011.226340 PMC3382329

[31] McEvoy, C. T. et al. Vitamin C supplementation for pregnant smoking women and pulmonary function in their newborn infants: a randomized clinical trial. *J. Am. Med. Assoc.* **311**, 2074–2082 (2014). 10.1001/jama.2014.5217 PMC4296045 24838476

[32] McEvoy, C. T. et al. Oral vitamin C (500 Mg/D) to pregnant smokers improves infant airway function at 3 months (Vcsip). a randomized trial. *Am. J. Respir. Crit. Care Med.* **199**, 1139–1147 (2019). 30522343 10.1164/rccm.201805-1011OC PMC6515875

[33] McEvoy, C. T. et al. Vitamin C to Pregnant Smokers Persistently Improves Infant Airway Function to 12 Months of Age: A Randomised Trial. *European Respiratory Journal* **56** (2020). 10.1183/13993003.02208-2019 PMC8029653 32616589

[34] Shorey-Kendrick, L. E. et al. Impact of vitamin C supplementation on placental DNA methylation changes related to maternal smoking: association with gene expression and respiratory outcomes. *Clin. Epigenetics* **13**, 1–17 (2021). 34538263 10.1186/s13148-021-01161-y PMC8451157

[35] Miller, S. L., Loose, J. M., Jenkin, G. & Wallace, E. M. The effects of sildenafil citrate (viagra) on uterine blood flow and well being in the intrauterine growth-restricted fetus. *Am. J. Obstet. Gynecol.* **200**, 102. e101–102. e107 (2009). 10.1016/j.ajog.2008.08.029 18845296

[36] Inocencio, I. M. et al. Effects of maternal sildenafil treatment on vascular function in growth-restricted fetal sheep. *Arteriosclerosis, Thrombosis, Vasc. Biol.* **39**, 731–740 (2019). 10.1161/ATVBAHA.119.312366 30841708

[37] Orgeig, S. & Morrison, J. L. Does the intrauterine growth-restricted fetus benefit from antenatal glucocorticoids? *Expert Rev. Obstet. Gynecol.* **5**, 149–152 (2010).

[38] Sharp, A., Cornforth, C. & Alfirevic, Z. Learning from the strider trial. *Obstet., Gynaecol. Reprod. Med.* **29**, 240–241 (2019).

[39] Morrison, J. L. et al. MRI characterization of blood flow and oxygen delivery in the fetal sheep whilst exposed to sildenafil citrate. *Neonatology* **119**, 735–744 (2022). 36252551 10.1159/000526972

[40] Gonzalez-Candia, A. et al. Antenatal melatonin modulates an enhanced antioxidant/pro-oxidant ratio in pulmonary hypertensive newborn sheep. *Redox Biol.* **22**, 101128 (2019). 30771751 10.1016/j.redox.2019.101128 PMC6375064

[41] Hansell, J. A. et al. Maternal melatonin: effective intervention against developmental programming of cardiovascular dysfunction in adult offspring of complicated pregnancy. *J. Pineal Res.* **72**, e12766 (2022). 34634151 10.1111/jpi.12766

[42] Richter, H. G., Hansell, J. A., Raut, S. & Giussani, D. A. Melatonin improves placental efficiency and birth weight and increases the placental expression of antioxidant enzymes in undernourished pregnancy. *J. Pineal Res.* **46**, 357–364 (2009). 19552758 10.1111/j.1600-079X.2009.00671.x

[43] Smith, K. L. et al. Chronic developmental hypoxia alters mitochondrial oxidative capacity and reactive oxygen species production in the fetal rat heart in a sex‐dependent manner. *J. Pineal Res.* **73**, e12821 (2022). 35941749 10.1111/jpi.12821 PMC9540814

[44] McGillick, E. V. et al. Maternal chronic hypoxia increases expression of genes regulating lung liquid movement and surfactant maturation in male fetuses in late gestation. *J. Physiol.* **595**, 4329–4350 (2017). 28318025 10.1113/JP273842 PMC5491863

[45] McGillick, E. V. et al. Molecular regulation of lung maturation in near-term fetal sheep by maternal daily vitamin C treatment in late gestation. *Pediatr. Res.* **91**, 828–838 (2022). 33859366 10.1038/s41390-021-01489-4 PMC9064793

[46] Morrison, J. L. et al. Improving pregnancy outcomes in humans through studies in sheep. *Am. J. Physiol.-Regulatory, Integr. Comp. Physiol.* **315**, R1123–R1153 (2018). 10.1152/ajpregu.00391.2017 30325659

[47] Allison, B. et al. Fetal in vivo continuous cardiovascular function during chronic hypoxia. *J. Physiol.* **594**, 1247–1264 (2016). 26926316 10.1113/JP271091 PMC4771786

[48] Brain, K. et al. Induction of controlled hypoxic pregnancy in large mammalian species. *Physiological Rep.* **3**, e12614 (2015). 10.14814/phy2.12614 PMC4760453 26660546

[49] McGillick, E. V. et al. Chronic fetal hypoxia and antenatal Vitamin C exposure differentially regulate molecular signalling in the lung of female lambs in early adulthood. *Front. Physiol.* **15**, 1488152 (2025). 10.3389/fphys.2024.1488152 PMC11775154 39882327

[50] Lock, M., McGillick, E. V., Orgeig, S., McMillen, I. C. & Morrison, J. L. Regulation of fetal lung development in response to maternal overnutrition. *Clin. Exp. Pharmacol. Physiol.* **40**, 803–816 (2013). 24033542 10.1111/1440-1681.12166

[51] Harding, R. & Bocking, A. D. *Fetal Growth and Development* (Cambridge Univ Pr, 2001).

[52] Sales, F. et al. Maternal supplementation with antioxidant vitamins in sheep results in increased transfer to the fetus and improvement of fetal antioxidant status and development. *Antioxidants* **8**, 59 (2019). 30857206 10.3390/antiox8030059 PMC6466585

[53] Thakor, A. et al. Redox modulation of the fetal cardiovascular defence to hypoxaemia. *J. Physiol.* **588**, 4235–4247 (2010). 20807788 10.1113/jphysiol.2010.196402 PMC3002453

[54] Niu, Y., Herrera, E. A., Evans, R. D. & Giussani, D. A. Antioxidant treatment improves neonatal survival and prevents impaired cardiac function at adulthood following neonatal glucocorticoid therapy. *J. Physiol.* **591**, 5083–5093 (2013). 23940378 10.1113/jphysiol.2013.258210 PMC3810811

[55] Herrera, E. A., Verkerk, M. M., Derks, J. B. & Giussani, D. A. Antioxidant treatment alters peripheral vascular dysfunction induced by postnatal glucocorticoid therapy in rats. *PLoS One* **5**, e9250 (2010). 20174656 10.1371/journal.pone.0009250 PMC2822858

[56] Thakor, A. S., Herrera, E. A., Serón‐Ferré, M. & Giussani, D. A. Melatonin and vitamin C increase umbilical blood flow via nitric oxide‐dependent mechanisms. *J. Pineal Res.* **49**, 399–406 (2010). 20958954 10.1111/j.1600-079X.2010.00813.x

[57] Jackson, T. S., Xu, A., Vita, J. A. & Keaney, J. F. Jr Ascorbate prevents the interaction of superoxide and nitric oxide only at very high physiological concentrations. *Circ. Res.* **83**, 916–922 (1998). 9797340 10.1161/01.res.83.9.916

[58] Russell, W. M. S. & Burch, R. L. *The Principles of Humane Experimental Technique* (Methuen, 1959).

[59] Vuilleumier, J. & Keck, E. Fluorometric Assay of Vitamin C in Biological Materials Using a Centrifugal Analyser with Fluorescence Attachment. *Journal of Micronutrient Analysis* (1989).

[60] Soo, P. S. et al. Maternal Undernutrition Reduces P-Glycoprotein in Guinea Pig Placenta and Developing Brain in Late Gestation. *Reproductive Toxicology*, 374-381 (2012). 10.1016/j.reprotox.2012.01.013 22326852

[61] Lie, S. et al. Impact of embryo number and maternal undernutrition around the time of conception on insulin signaling and gluconeogenic factors and micrornas in the liver of fetal sheep. *Am. J. Physiol.-Endocrinol. Metab.* **306**, E1013–E1024 (2014). 24496309 10.1152/ajpendo.00553.2013 PMC4010656

[62] McGillick, E. V., Orgeig, S., McMillen, I. C. & Morrison, J. L. The fetal sheep lung does not respond to cortisol infusion during the late canalicular phase of development. *Physiol. Rep.* **1**, e00130 (2013). 24400136 10.1002/phy2.130 PMC3871449

[63] Bustin, S. A. et al. The Miqe guidelines: minimum information for publication of quantitative real-time PCR experiments. *Clin. Chem.* **55**, 611–622 (2009). 19246619 10.1373/clinchem.2008.112797

[64] Keller-Wood, M., von Reitzenstein, M. & McCartney, J. Is the fetal lung a mineralocorticoid receptor target organ? induction of cortisol-regulated genes in the ovine fetal lung, kidney and small intestine. *Neonatology* **95**, 47–60 (2009). 18787337 10.1159/000151755 PMC2654587

[65] Botting, K. J., McMillen, I. C., Forbes, H., Nyengaard, J. R. & Morrison, J. L. Chronic hypoxemia in late gestation decreases cardiomyocyte number but does not change expression of hypoxia-responsive genes. *J. Am. Heart Assoc.* **3**, e000531 (2014). 25085511 10.1161/JAHA.113.000531 PMC4310356

[66] Orgeig, S., Crittenden, T. A., Marchant, C., McMillen, I. C. & Morrison, J. L. Intrauterine growth restriction delays surfactant protein maturation in the sheep fetus. *Am. J. Physiol.-Lung Cell. Mol. Physiol.* **298**, L575–L583 (2010). 20097737 10.1152/ajplung.00226.2009

[67] Zhang, S. et al. Early restriction of placental growth results in placental structural and gene expression changes in late gestation independent of fetal hypoxemia. *Physiol. Rep.* **4**, e13049 (2016). 27923976 10.14814/phy2.13049 PMC5357827

[68] Wang, K. C. et al. Alteration of cardiac glucose metabolism in association to low birth weight: experimental evidence in lambs with left ventricular hypertrophy. *Metabolism* **62**, 1662–1672 (2013). 23928106 10.1016/j.metabol.2013.06.013

[69] Lock, M. C. et al. Mature surfactant protein-b expression by immunohistochemistry as a marker for surfactant system development in the fetal sheep lung. *J. Histochem. Cytochem.* **63**, 866–878 (2015). 26297137 10.1369/0022155415600201 PMC4812676

[70] Polglase, G. R. et al. Effects of antenatal melatonin therapy on lung structure in growth-restricted newborn lambs. *J. Appl. Physiol.* **123**, 1195–1203 (2017). 28819007 10.1152/japplphysiol.00783.2016

[71] Bartman, C. M., Awari, D. W., Pabelick, C. M. & Prakash, Y. Intermittent hypoxia-hyperoxia and oxidative stress in developing human airway smooth muscle. *Antioxidants* **10**, 1400 (2021). 34573032 10.3390/antiox10091400 PMC8467919

[72] Wiese, M. D., Berry, M. J., Hissaria, P., Darby, J. R. & Morrison, J. L. Covid-19: can we treat the mother without harming her baby? *J. Develop. Orig. Health Dis.* **13**, 9–19 (2022). 10.1017/S2040174420001403 33487213

[73] Soo, J. Y., Wiese, M. D., Berry, M. J. & Morrison, J. L. Does poor fetal growth influence the extent of fetal exposure to maternal medications? *Pharmacol. Res.* **130**, 74–84 (2018). 29425726 10.1016/j.phrs.2018.02.001

[74] González-Candia, A. et al. Potential adverse effects of antenatal melatonin as a treatment for intrauterine growth restriction: findings in pregnant sheep. *Am. J. Obstet. Gynecol.* **215**, 245. e241–245. e247 (2016). 10.1016/j.ajog.2016.02.040 26902986

[75] Lee, J. Y. et al. Melatonin for prevention of fetal lung injury associated with intrauterine inflammation and for improvement of lung maturation. *J. Pineal Res.* **69**, e12687 (2020). 32737901 10.1111/jpi.12687

[76] Vento, M. et al. Antenatal steroids and antioxidant enzyme activity in preterm infants: influence of gender and timing. *Antioxid. Redox Signal.* **11**, 2945–2955 (2009). 19645572 10.1089/ars.2009.2671

